# Alterations in Amygdala Connectivity in Internet Addiction Disorder

**DOI:** 10.1038/s41598-020-59195-w

**Published:** 2020-02-11

**Authors:** Hewei Cheng, Jie Liu

**Affiliations:** 1 Department of Biomedical Engineering, School of Bioinformatics, Chongqing University of Posts and Telecommunications, Chongqing, China; 2 Department of Radiology, Perelman School of Medicine, University of Pennsylvania, Philadelphia, USA; 3 Research Institute of Education Development, Chongqing University of Posts and Telecommunications, Chongqing, China

**Keywords:** Neuroscience, Signs and symptoms

## Abstract

Recent studies have revealed structural and functional abnormalities in amygdala due to Internet addiction (IA) associated with emotional disturbance. However, the role of amygdala connectivity that is responsible for emotion-cognition interactions is largely unknown in IA. This study aims to explore the amygdala connectivity abnormalities in IA. The functional and structural connectivity of bilateral amygdala were examined using seed-based connectivity analysis, and the structural integrity on white mater tracts passing through amygdala was also examined. Additionally, a correlation analysis was performed to investigate the relationship between brain connectivity and duration of IA. We found that IA subjects had decreased negative functional connectivity (FC) between amygdala and dorsolateral prefrontal cortex (DLPFC), and had increased negative FC between amygdala and precuneus and superior occipital gyrus (SOG). While IA subjects had decreased positive FC between amygdala and anterior cingulate cortex (ACC), and had increased positive FC between amygdala and thalamus. The FC between left amygdala and right DLPFC had significant correlation with duration of IA. The structural connectivity and integrity between amygdala and ACC were also decreased in IA subjects. These findings indicate that the amygdala connectivity is altered in IA subjects. The altered FC of amygdala-DLPFC is associated with duration of IA.

## Introduction

Internet addiction (IA) is a behavioral addiction, which has become a social problem across Eastern and Western societies with a prevalence rate ranging from 1.4% to 20.3%^1^. More importantly, the prevalence rate of IA among adolescents has an increasing trend^1,2^. IA is generally characterized by a loss of control over the use of the Internet despite its adverse consequences^3^. The excessive and compulsive Internet use has accounted for poor real-life performance, affecting daily life function, social relationships, and academic or occupational achievements^4,5^. Empirical evidence is accumulating that IA subjects have altered cognitive behaviors and brain structures^6–8^.

Clinically, IA disorder is closely associated with other psychiatric disorders, such as substance use disorder^9,10^, depression^10^, social anxiety disorder^11^, and attention-deficit hyperactivity disorder^12^. Particularly, IA shares some clinical symptoms with them, such as preoccupation, withdrawal, negative mood, and unsuccessful attempts to control^3,13^. The similar emotional and cognitive disturbances suggest overlaps in the neurobiological mechanisms underlying IA and these psychiatric disorders.

A number of neuroimaging studies have documented that the amygdala played an important role in emotion processing and involved in psychiatric disorders, such as IA associated with emotional disturbance^14–17^. In particular, a neuroimaging study investigated the brain grey matter volume using voxel-based morphometry (VBM) and found that the grey matter volume in the amygdala was decreased in IA^14^. A resting-state functional magnetic resonance imaging (rsfMRI) study reported that IA subjects showed reduced regional centrality in the amygdala^15^. An emotion-task fMRI study reported that the amygdala was abnormally activated in IA^17^. In addition, another neuroimaging study investigated the brain metabolism of IA subjects using arterial spin labeled perfusion magnetic resonance imaging and found that IA subjects had abnormally high metabolic rate in the amygdala^16^. These neuroimaging studies have demonstrated that the amygdala was functionally and structurally changed in IA.

Additionally, amygdala and prefrontal cortex (PFC) constitute the key circuit for emotion-cognition interactions^18^ and contributes to the pathogenesis of affective and addictive disorders^19–24^. The amygdala-PFC functional coupling can be used to distinguish individuals with different abilities for cognitively regulating negative emotions^25^. Specifically, the amygdala-PFC connectivity is responsible for the regulation of emotional status (irritability, anxiety, and aversive stress-like state), which is associated with negative reinforcement and motivational effects in drug addiction, and impaired connectivity could result in perpetuated drug use or relapse^22,26^. Decreased resting-state functional connectivity (rsFC) between amygdala and medial PFC in both active cocaine and heroin users has been reported^27,28^. Opioid-dependent patients have lower fractional anisotropy (FA) in the primary white matter tract traversing amygdala and PFC^29,30^. Moreover, several studies have reported abnormal craving-related activity in brain regions distributed over the brain in IA subjects, such as dorsolateral PFC (DLPFC), anterior cingulate cortex (ACC), thalamus, precuneus and superior occipital gyrus (SOG)^31–36^. All these findings indicate that abnormal amygdala connectivity might impair self-control ability critical for craving regulation and play important roles in the onset and maintenance of IA.

In this study, we hypothesized that the amygdala connectivity might be altered in IA subjects and subsequently affect their affective and cognitive abilities. Using resting-state functional magnetic resonance imaging and diffusion tension imaging (DTI) data, amygdala seed-based resting-state functional connectivity analysis and fiber tractography were performed respectively to explore the amygdala functional and structural connectivity in IA. Furthermore, correlation analysis was conducted between the brain connectivity measures and the duration of IA.

## Results

### Subject characteristics

The demographic, behavioral and psychological measures for IA and healthy control (HC) subjects are summarized in Table 1. In the IA group, the mean duration of IA was estimated to be 27.8 months. Subjects with IA spent significantly more time on the Internet daily than healthy controls (HCs) (*p* = 0.000). Self-reported levels of anxiety and depression were significantly higher in IA subjects compared with HCs (*p* = 0.015 & 0.000, respectively). The IA subjects had weaker self-control ability than HCs (*p* = 0.007). For IA subjects, self-reported levels of anxiety and depression were significantly correlated (*p* = 0.001), and none of them were significantly correlated with self-control ability (*p* = 0.120 & 0.057, respectively).

**Table 1:** Demographic data, behavioral and psychometric measurements of participants.

Characteristics	IA subjects (n = 24; Mean ± SD)	HCs (n = 28; Mean ± SD)	p value
Age (years)	20.7 ± 1.9	21.0 ± 1.6	0.540
Sex (Male/Female)	18/6	22/6	0.761
Duration of Internet addiction (months)	27.8 ± 11.6		
Average online time per day (hours)	9.8 ± 3.9	2.2 ± 1.5	0.000
Self-control (Cn)	23.9 ± 6.9	28.9 ± 5.9	0.007
Anxiety (HAMA)	11.4 ± 8.4	6.6 ± 3.3	0.015
Depression (BDI-13)	9.4 ± 5.7	3.6 ± 2.3	0.000

IA, Internet Addiction; HCs, Healthy Controls; SD, Standard Deviation; Cn, Control Scale in Minnesota Multiphasic Personality Inventory; HAMA, Hamilton Anxiety Scale; BDI-13, Beck Depression Inventory (13 items).

### Abnormal functional connectivity of amygdala in IA

**Figure 1:**
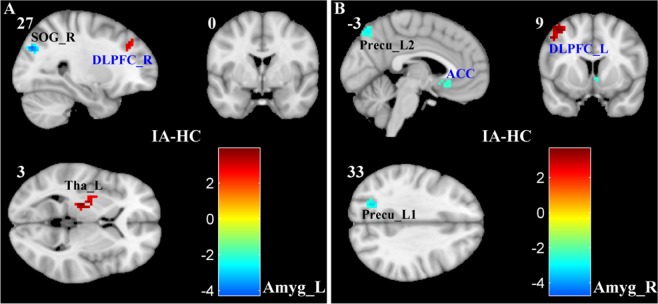
Altered functional connectivity between amygdala and prefrontal cortex (PFC) in subjects with IA. Statistically significant group differences in the amygdala-seeded rsFC measure between IA and HC groups were identified using two-sample t-tests at a threshold of p < 0.05 using false discovery rate correction for multiple comparisons. The colorbar indicates *t* values of two-sample t-tests. Abbreviations: rsFC, resting state functional connectivity; IA, Internet addiction; HC, healthy control; Amyg, amygdala; L, left; R, right. The other abbreviations are given in Table 2. The figure was drawn by using xjView (xjView version 9.6) (https://www.alivelearn.net/xjview/).

**Figure 2:**
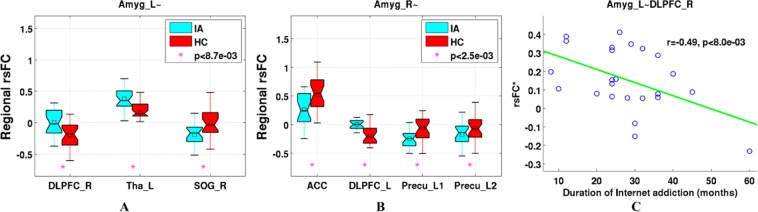
Relationship between regional rsFC of amygdala-PFC and duration of IA. (**A**,**B**) Regional rsFC measures between amygdala and brain regions of PFC shown in Fig. 1, and all the regional rsFC had statistically significant difference between IA and HC groups with *p* < 8.7e-03 and *p* < 2.5e-03 for left and right amygdala, respectively. (**C**) The negative correlation between the adjusted regional rsFC measure of left amygdala-DLPFC_R and the duration of Internet addiction (months) in the IA group(*r* = −0.49, *p* < 8.0e-03). The rsFC* is the *z* value of regional rsFC measure adjusted by age, sex, HAMA score and BDI-13 score using multiple linear regression. In (**A**,**B**), on each box, the central mark is the median, the edges of the box are the 25th and 75th percentiles, and whiskers extend from each edge of the box to the most extreme values within 1.5 times the interquartile range. Stars indicate that the difference was statistically significant, and *p* values were results from two-sample t-test. Abbreviations: HAMA, Hamilton Anxiety Scale; BDI-13, Beck Depression Inventory (13 items). The other abbreviations are given in Fig. 1 and Table 2. The figure was drawn by using MATLAB (R2016b version 9.1.0.441655) (https://www.mathworks.com/).

Several brain regions of PFC had altered rsFC with the amygdala, including DLPFC and ACC. Particularly, compared with HCs, the IA subjects had significantly decreased negative rsFC for connections between left amygdala and right middle frontal gyrus (Brodmann area 9 and 8, DLPFC), and between right amygdala and left middle frontal gyrus (Brodmann area 8, DLPFC and Brodmann area 6, premotor cortex) (Figs. 1 and 2A,B and Table 2). The positive rsFC of connection between right amygdala and bilateral ACC (Brodmann area 25) was also decreased in the IA group (Figs. 1 and 2B and Table 2). Furthermore, the IA subjects had increased positive rsFC of connection between left amygdala and left thalamus, and increased negative rsFC for connections between left amygdala and right superior occipital gyrus, and between right amygdala and left precuneus regions (Figs. 1 and 2A,B and Table 2).

**Table 2:** Regions with statistically significant differences between IA subjects and HCs in the functional connectivity with amygdala.

Seed	Regions of difference (Brodmann area)	Cluster size (voxels)	Peak MNI coordinate	Peak intensity (*z* value)
Left amygdala	right middle frontal gyrus(9/8)^a^	40	30, 36, 39	2.30
	left thalamus^b^	44	−21, −21, 3	3.62
	right superior occipital gyrus (19)^c^	39	27, −78, 33	−3.86
Right amygdala	bilateral anterior cingulate cortex (25)^d^	25	3, 18, −15	−3.04
	left middle frontal gyrus (8/6)^e^	53	−42, 12, 51	3.40
	left precuneus (7)^f^	92	−24, −66, 27	−4.21
	left precuneus(7)^g^	47	−6, −72, 51	−3.05

IA, Internet Addiction; HCs, Healthy Controls; MNI, Montreal Neurological Institute. Corresponding abbreviations in Fig. 1 and Fig. 2: ^a^DLPFC_R; ^b^Tha_L; ^c^SOG_R; ^d^ACC; ^e^DLPFC_L; ^f^Precu_L1; ^g^Precu_L2.

### Abnormal structural connectivity of amygdala in IA

**Figure 3:**
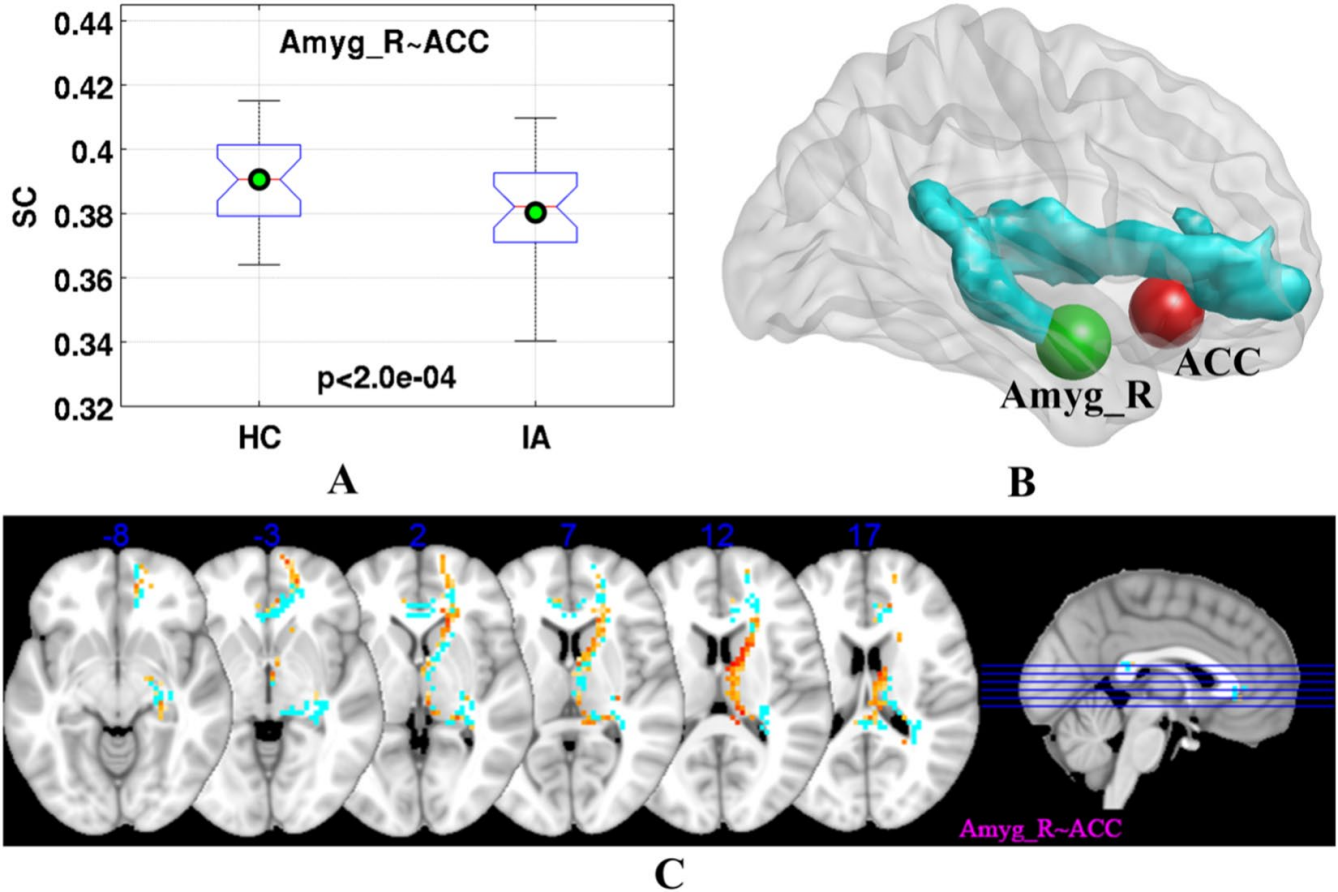
Altered structural connectivity (SC) and fractional anisotropy (FA) in subjects with IA. (**A**) SC measures of the pathway between right amygdala and ACC, and the differences in SC measures between HC and IA groups were statistically significant (*p* < 2.0e-04). (**B**) 3D view of the white matter pathway traversing right amygdala and ACC (in cyan). (**C**) Brain regions (in red/yellow) with statistically significant difference in FA between IA and HC groups on the white matter pathway traversing right amygdala and ACC (in cyan). In (**A**), on each box, the central mark is the median, the edges of the box are the 25th and 75th percentiles, and whiskers extend from each edge of the box to the most extreme values within 1.5 times the interquartile range. The *p* values were results from two-sample t-test. The abbreviations are given in Fig. 1 and Table 2. The (**A**–**C**) were drawn by using MATLAB (R2016b version 9.1.0.441655) (https://www.mathworks.com/), BrainNet Viewer (BrainNet Viewer 1.7) (https://www.nitrc.org/projects/bnv/), and MRIcron (MRIcron version 1.0.20190902) (https://www.nitrc.org/projects/mricron/), respectively.

Among the white matter tracts traversing the amygdala and brain regions with altered functional connectivity, the white matter pathway traversing the right amygdala and ACC had significantly decreased structural connectivity (SC) in IA subjects (Fig. 3A,B *p* < 2.0e-04), and voxels with significantly decreased fractional anisotropy (FA) were distributed along the path (Fig. 3C). This white matter tract traversing the right amygdala and ACC followed the pathway posterior limb of internal capsule-cerebral peduncle-retrolenticular part of internal capsule.

### Correlation between brain connectivity and duration of IA

A negative correlation with statistical significance was observed between the duration of IA and the rsFC of connection between the left amygdala and right DLPFC (Fig. 2C, *p* < 8.0e-03).

## Discussion

In this study, we examined the amygdala functional and structural connectivity in IA subjects. The rsFC of amygdala to brain regions, including DLPFC, ACC, thalamus, precuneus and SOG, was altered due to IA. Particularly, IA subjects had reduced negative rsFC between amygdala and DLPFC, and had elevated negative rsFC between amygdala and precuneus and SOG. In addition, IA subjects had reduced positive rsFC between amygdala and ACC, and had elevated positive functional connectivity between amygdala and thalamus. Importantly, the rsFC between amygdala and DLPFC was significantly correlated with the duration of IA. The SC between the amygdala and ACC was also altered due to IA.

Behavioral results indicated that IA subjects had low self-control ability for controlling their excessive Internet use, and had subclinical symptoms including anxiety and depression. The two subclinical symptoms of these IA subjects had close relationship measured by the correlation between the self-reported levels of anxiety and depression. Dysfunction in the amygdala-PFC circuit might cause these subclinical symptoms and psychological frustration accompanying social defeat and withdrawal and potentially contributing to the development of IA, or vice versa. The dynamic interplay between amygdala and PFC enables individuals to react to salient stimuli and regulates emotional responses to adapt to different situations^37,38^. This cognitive ability to self-regulate emotions is a crucial skill for psychological well-being, resilience, and proper social interaction^39^. The structural features of amygdala and PFC have been correlated with the size and complexity of social networks in healthy individuals^40^. Studies have shown that the IA subjects typically presented low interpersonal skills, lacking of the sense of social belonging, and feelings of loneliness in the real life^41,42^. Therefore, these deficits in social functioning might be the consequence of the disrupted amygdala-PFC connectivity found in this study, or vice versa.

Excessive Internet use is highly prevalent among adolescents^1,2^, who are in a critical period for the amygdala-PFC circuit to mature and build up the emotional regulatory ability^43,44^. Dealing with everyday distress is a great challenge for the developing emotional circuitry. Individual differences in the use of emotional regulatory strategies have been used to predict amygdala and PFC responses to emotional challenges^45^. In subjects with IA, addicting to Internet has become a favorable way of escaping from stressful situations and unpleasant feelings^13^. This deficiency of applying effective coping strategies under stressful situations might correspond to underdeveloped amygdala-PFC connectivity in IA subjects. However, the causal relationship between neural abnormalities and emotional dysregulation cannot be inferred yet from the present study.

The DLPFC in PFC has been suggested to exert high-level cognitive control over the amygdala response in the context of emotional regulation^46,47^. The ACC in PFC had the densest structural connections with amygdala^48^. This brain region has been implicated in a validated amygdala network involved in emotion processing^49^, including the integration of emotional significance of stimuli, generation of emotional states, and cognitive emotional regulation^46,50^. These findings indicate that connections of amygdala-PFC (DLPFC/ACC), the negative and positive functional connections for amygdala-DLPFC and amygdala-ACC respectively^51^, play different roles in emotional regulation for development of IA.

We found that the negative amygdala-DLPFC functional connectivity was decreased due to IA. One of the major emotional regulation challenges facing IA subjects is the attempt to control the craving for Internet use, which might require the neural dynamics in the amygdala-PFC circuit. Subjects with IA have excessive Internet use, which might reflect on the failure of craving control. Previous reports have shown craving-related activity in DLPFC in IA subjects^31,33^. A previous study had reported that the positive functional connectivity of amygdala-DLPFC was decreased in IA subjects^14^. These findings might indicate that DLPFC has heterogeneous functional subregions responsible for different cognitive processes^52^.

Our analysis also revealed a significant correlation between the altered amygdala-DLPFC functional connectivity and the durations of IA. It has been suggested that the dysregulation of amygdala-PFC circuit persists through protracted abstinence, which makes individuals vulnerable to craving and relapse in drug addiction^26^. For IA subjects, since much comfort as the stress-induced Internet use can provide temporarily, excessive involvement in online activities could result in further distress, which makes it even harder to control the Internet use. The amygdala-DLPFC connectivity might suffer a progressive damage during the development of IA. Therefore, additional deficit in this connectivity would occur as the duration of excessive Internet use prolonged.

The positive functional connectivity between amygdala and ACC was also decreased due to IA. Deficits in the implicit regulation of emotional processing have been associated with insufficient ACC activation and connectivity with the amygdala^19^. The abnormal amygdala-ACC connectivity might contribute to the emotional dysregulation associated with IA.

We also found abnormal structural connectivity of amygdala-PFC (ACC) in IA subjects, along with decreased structural integrity on the white matter pathway. These structural abnormalities could constitute the neuroanatomical basis for the functional deficits in IA subjects. These findings might also suggest an interaction between the maturation process of amygdala-PFC circuit and Internet use behavior. The developing amygdala-PFC connectivity might underlie the vulnerability of adolescents to IA, and persistence of maladaptive Internet use behavior might in turn interfere with the maturation of PFC and its regulatory function. The disrupted formation of appropriate neural circuitry would then result in deficiency of affective and cognitive abilities required for proper decision-making and social behavior. Gradually, a vicious cycle might emerge between IA and dysregulation of amygdala-PFC circuit.

Additionally, abnormal functional connectivity in IA subjects between amygdala and other brain regions was also observed, including thalamus, precuneus, and SOG (Figs. 1, 2A,B and Table 2). These regions have shown increased activity when IA subjects craved for Internet use^32,33,36^. The thalamus had direct structural connections with the amygdala, and these connections have been implicated in reacting to salient stimuli and processing emotion responses^53^. The abnormal functional connectivity of amygdala-thalamus might also contribute to the emotional dysregulation associated with IA. The precuneus involved in processing visual spatial information and drawing attention to external environment, and the functional connectivity between amygdala and precuneus participated in regulating emotion during attentional deployment^54,55^. The elevated negative functional connectivity between amygdala and precuneus might be to adapt and satisfy high visual attention loads during persistent and excessive Internet use in IA subjects. The third brain region, i.e., SOG, lies in the occipital lobe belonging to the visual cortex, which exhibited relatively higher activations when responded to targets presented in the near space than in the far space^56^. The increased negative functional connectivity between amygdala and SOG might trigger adaptation for excessive near-space visual search on the computer screen during permanent Internet use in IA subjects.

In this study, the rsfMRI data was preprocessed without the global signal regression. However, as shown in Supplementary Fig. S1 (left) versus Supplementary Fig. S1 (right) and Supplementary Fig. S2A,B versus Fig. 2A,B, the general pattern of amygdala functional connectivity obtained from data preprocessed without the global signal regression was similar to those obtained from the data processed with the global signal regression, indicating that the findings of our study were stable with respect to the data preprocessing^57^.

In conclusion, the altered functional and structural connectivity between amygdala and PFC in individuals with abnormal Internet use behavior could have a major influence on the development of IA, probably through its close relationship with the self-regulation of emotions. The connectivity of amygdala-PFC (DLPFC/ACC) might be progressively damaged in the development of IA. These findings shed light on the importance of amygdala-PFC interactions in the neural mechanisms of IA and provided specific targets for clinical interventions.

## Limitations

There are several limitations in the present study. Firstly, the recruited IA subjects in this study are undergraduates, which might not be representative for the population of persons with Internet addiction. Secondly, we did not study subregional connections of the amygdala due to the relatively low spatial resolution of our fMRI data. Thirdly, our DTI data is anisotropic resolution, which might produce biased estimates for whiter matter tracts and fractional anisotropy. Furthermore, the amygdala is susceptible to image distortions, normalization errors, and draining vein effects which may lead to spatial localization errors^58,59^.

## Methods

### Participants

Twenty four freshmen and sophomore students with IA (18 males, age: 20.7 ± 1.9 years) participated in this study. The 24 IA subjects were enrolled based on the modified Young Diagnostic Questionnaire (YDQ) criteria for IA^3^. The duration of IA was estimated via a retrospective diagnosis. The subjects were asked to recall their life-styles when they were initially addicted to the Internet. To guarantee that they were suffering from IA at that time, we retested them with the YDQ criteria modified by Beard and Wolf^3^. The reliability of self-reports from the IA subjects was confirmed by interviewing with their parents, and it was also verified from their roommates and classmates that they often insisted on staying on the Internet late at night despite the negative life consequences. Twenty eight age- and sex-matched freshmen and sophomore students (healthy controls, HCs) (22 males, age: 21.0 ± 1.6 years) with no personal or family history of psychiatric disorders also participated in this study.

This study was carried out in accordance with the Code of Ethics of the World Medical Association (Declaration of Helsinki) and approved by the Institutional Ethics Committee of Biomedical Engineering, Chongqing University of Posts and Telecommunications, Chongqing, China. Written informed consent was obtained from all participants.

### Psychometric measurements

To assess the psychological features of the participants, certain self-rating questionnaires were completed by all participants. Thirteen-item Beck Depression Inventory (BDI-13)^60^ and Hamilton Anxiety Scale (HAMA)^61^ were used to evaluate levels of depression and anxiety, respectively. The self-control ability was estimated by the control scale in the Minnesota Multiphasic Personality Inventory^62^.

### Imaging data acquisition

Resting-state fMRI data was acquired with 180 volumes using a gradient echo-planar imaging sequence, and the imaging parameters were 32 transverse slices with no gap, slice thickness = 4 mm, in-plane resolution = 3.75 × 3.75 mm^2^, field of view [FOV] = 240 × 240 mm^2^, repetition time [TR] = 2000 ms, echo time [TE] = 30 ms, and flip angle = 90°. DTI data was collected with 25 non-linear directions (b = 1000 s/mm^2^) together with an acquisition without diffusion weighting (b = 0 s/mm^2^). The imaging parameters were 33 axial slices with no gap, slice thickness = 4 mm, in-plane resolution = 0.94 × 0.94 mm^2^, FOV = 240 × 240 mm^2^, TR = 7600 ms, and TE = 72.5 ms. In addition, structural 3D T1-weighted images were collected with a spoiled gradient recall sequence, and the imaging parameters were 166 axial slices with no gap, slice thickness = 1 mm, in-plane resolution = 1 × 1 mm^2^, FOV = 256 × 256 mm^2^, TR = 1900 ms, TE = 2.26 ms, and flip angle = 90°.

### Image data preprocessing

#### Resting-state fMRI data preprocessing

The fMRI data was pre-processed as follows: (1) discarding the first 6 time points; (2) slice-timing correction and head motion correction; (3) intensity scaling of the fMRI data to yield a whole-brain mean value of 10000; (4) regressing out of nuisance signals including averaged signals of the white matter and the cerebrospinal fluid regions, and 6 affine motion parameters; (5) temporally band-pass filtering (0.01 < *f* < 0.08 Hz); (6) normalizing the fMRI data nonlinearly into Montreal Neurological Institute (MNI) space with the deformation fields of their co-registered T1-weighted images using DARTEL within SPM12, and resampling the fMRI data to resolution 3 × 3 × 3 mm^3^; (7) spatially smoothing with a 6 mm full width at half maximum (FWHM) Gaussian kernel. All these fMRI data preprocessing procedures were performed using SPM12 (Statistical Parametric Mapping, SPM12 version 7487) (https://www.fil.ion.ucl.ac.uk/spm/software/spm12/).

The head motion of fMRI data was measured by the maximum displacement for brain voxels between two successive time points was no more than 1 mm, and the root-mean-square value of the frame-wise displacement was no more than 1.5 mm^63^.

#### DTI data preprocessing

The DTI data was pre-processed using steps: (1) DTI data were corrected for eddy currents and head motion by affine registration to the non-weighted b0 image; (2) the skull was subsequently removed; (3) fractional anisotropy was calculated based on the skull removed data for group analysis. All these steps were performed using FSL^64,65^ (FMRIB Software Library, FSL version 5.0.9) (https://fsl.fmrib.ox.ac.uk/fsl/fslwiki/FslInstallation/). For each subject, the normalization of results calculated in DTI space to MNI space was implemented using DARTEL of spm12 with the deformation fields of the DTI data’s co-registered T1-weighted image (Statistical Parametric Mapping, SPM12 version 7487) (https://www.fil.ion.ucl.ac.uk/spm/software/spm12/).

### Behavioral data analysis

Demographic data and psychometric measurements were analyzed with two-sample t-test (Chi-Square test for sex distribution) between IA and HC groups using SPSS 16 (SPSS Inc., IL, USA). In addition, correlations between each pair of psychometric measurements, i.e., self-control, anxiety and depression, were analyzed by using Pearson correlation in the IA group.

### Imaging data analysis

#### Extraction of seed regions

To detect the brain functional characteristics underlying IA, we conducted seed-based functional connectivity analysis based on the resting-state fMRI data. Given the important role of amygdala-PFC circuit in affective and addictive disorders^19–24^, we chose amygdala as the seed for the connectivity analysis. For each subject, left and right amygdala, used as seed regions of interest (ROIs), were segmented from the T1-weighted image of the subject using Freesurfer^66^ (Freesurfer version stable v6.0.0) (http://www.freesurfer.net/).

#### Calculation of functional connectivity maps

For each subject and each seed ROI, the mean time series of voxels in the seed ROI was used as the seed signal for computing functional connectivity maps of the whole brain voxel-wise based on Pearson correlation^67^. The resulting whole brain voxel-wise Pearson correlation coefficients were then transformed to *z* values using Fisher’s *z* transform for subsequent group analysis^68^.

#### Group analysis of functional connectivity

Group analysis was performed on the functional connectivity maps (*z* values). The HAMA and BDI-13 scores were adopted as covariates for identifying group differences caused by Internet addcition alone rather than anxiety and depression. Particularly, for each seed ROI, one-sample t-test was applied voxel-wise to the whole brain functional connectivity maps of all the subjects with HAMA and BDI-13 scores as covariates in the HC group, and brain regions with statistically significant functional connectivity were identified at a threshold of *p* < 0.05 using false discovery rate (FDR) correction for multiple comparisons at the cluster level. For each seed ROI, two-sample t-test was applied voxel-wise to the functional connectivity maps with HAMA and BDI-13 scores as covariates between the two groups. The two-sample t-test was restricted to a mask consisting of voxels having statistically significant functional connectivity with the seed ROI in the HC group. Brain regions with statistically significant difference between IA and HC groups, referred to as regions of interest with altered functional connectivity (afc_ROIs), were identified at a threshold of *p* < 0.05 using FDR correction for multiple comparisons at the cluster level.

#### ROI-based white matter tractography analysis

For each subject, white matter tracts between seed and each target ROI were obtained by probabilistic tractography implemented using FSL with its default parameters (number of samples = 5,000, curvature threshold = 0.2, step length = 0.5 mm, number of steps = 2,000)^64,65^ (FMRIB Software Library, FSL version 5.0.9) (https://fsl.fmrib.ox.ac.uk/fsl/fslwiki/FslInstallation/), and spatially normalized to MNI space implemented using DARTEL of spm12 with the deformation fields of the DTI data’s co-registered T1-weighted image. The target ROI was the ROI with altered functional connectivity (i.e., afc_ROI) to the seed (amygdala) caused by IA described in “group analysis of functional connectivity”. Probabilistic tractography was performed with FSL’s “PROBTRACKX” to compute a probabilistic tract from each voxel of one ROI to the other ROI adopted as waypoint mask, and vise versa. Only streamlines that passed through the waypoint mask would be considered valid. Then, the probabilistic white matter tracts were tested voxel-wise between each pair of ROIs (i.e., amygdala and target ROI) across all IA and HC subjects using one-sample permutation test respectively, and white matter tracts with statistically significantly larger than zero were identified with *p* < 0.05 (threshold-free cluster enhancement and family-wise error rate corrected for multiple comparisons)^69^. The resulting white matter tracts were referred to as group-level white matter tracts.

#### Group analysis of structural connectivity

Group analysis was performed on the structural connectivity and structural integrity, respectively. The HAMA and BDI-13 scores were adopted as covariates for identifying group differences caused by Internet addiction alone rather than anxiety and depression. For each subject, the SC was measured by averaged FA values along the group-level whiter matter tracts traversing two brain regions^70^. Then, a two-sample permutation test was used to investigate whether the SC between amygdala and each of the afc_ROIs was different with HAMA and BDI-13 scores as covariates between IA and HC groups.

The structural integrity for voxels on group-level white matter tracts was examined voxel-wise by applying two-sample permutation test to their FA values with HAMA and BDI-13 scores as covariates between IA and HC groups. The brain regions on the white matter tracts with statistically significant differences in FA were identified with *p* < 0.05 (threshold-free cluster enhancement and family-wise error rate corrected for multiple comparisons)^69^.

#### Correlation analysis between brain connectivity and duration of Internet addiction

Correlation analysis was performed to explore the relationship between brain connectivity measures and duration of Internet addiction. Firstly, for each subject, rsFC and SC measures between amygdala and each of its afc_ROIs were obtained^68,70^. Then, the correlation between the duration of Internet addiction and each of the rsFC and SC measures was estimated by a general linear model with age, sex, HAMA score and BDI-13 score as covariates in the IA group using multiple linear regression. The statistical significant correlations were identified with *p* < 0.05 using FDR correction for the number of altered connections with the seed (i.e., amygdala).

## Supplementary information

Supplementary Information.
