# Supplementary material for: Alterations in Amygdala Connectivity in Internet Addiction Disorder
Source: Sci Rep. 2020 Feb 11;10:2370. doi: 10.1038/s41598-020-59195-w (PMC7012850; doi:10.1038/s41598-020-59195-w)
Supplement: Supplementary file 1 — Supplementary Information. [file 41598_2020_59195_MOESM1_ESM.pdf]

Supplementary materials for Scientific Reports

Alterations in Amygdala Connectivity in Internet Addiction Disorder

Hewei Cheng[1,2], Jie Liu[3,*]

[1]Department of Biomedical Engineering, School of Bioinformatics, Chongqing University of Posts and Telecommunications, Chongqing, China

[2]Department of Radiology, Perelman School of Medicine, University of Pennsylvania, Philadelphia, USA

[3]Research Institute of Education Development, Chongqing University of Posts and Telecommunications, Chongqing, China

*<u>Corresponding author:</u>

Jie Liu

Research Institute of Education Development

Chongqing University of Posts and Telecommunications

2, Chongwen Road, Nan'an District

Chongqing 400065, China

Email: jieliu218@163.com

Tel: +86-23-62483359

Effect of global signal regression on results of functional connectivity

The rsfMRI data was also pre-processed using the same protocol, but with the global signal regression.

Fig. S1 shows functional connectivity patterns of the amygdala for both HC and IA groups based on rsfMRI data pre-processed without (left) and with (right) the global signal regression, respectively. The results indicated that the functional connectivity patterns of amygdala were similar independent of global signal regression.

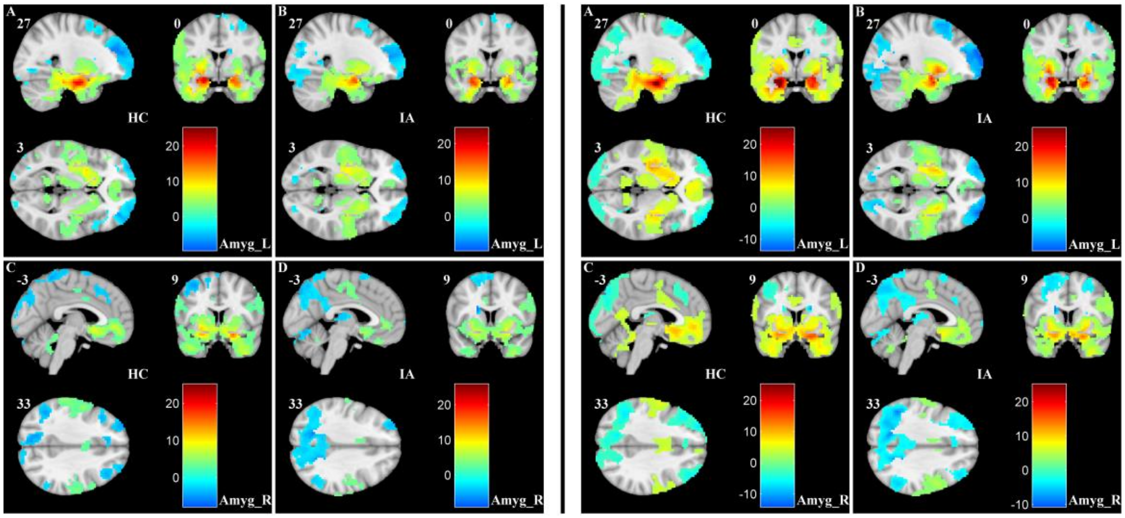

Supplementary Figure S1. Functional connectivity maps of amygdala in HC and IA groups obtained from rsfMRI data pre-processed without (left) and with (right) the global signal regression, respectively. Brain regions with statistically significant functional connectivity were identified at a threshold of $p<0.05$ using false discovery rate correction for multiple comparisons at the cluster level. Warm colors denote positive functional connectivity, and cold colors denote negative functional connectivity. The abbreviations are given in Fig.1.

Fig. 2S shows rsFC measures of connections between amygdala and brain regions of PFC shown in Fig. 1, computed based on the rsfMRI data pre-processed with the global signal regression. As shown in Fig. 2S(A and B), the rsFC measures obtained based on the rsfMRI data pre-processed with the global signal regression are nearly the same as those obtained based on the rsfMRI data pre-processed without the global signal regression shown in Fig. 2(A and B), indicating our findings were not affected by the data preprocessing procedure.

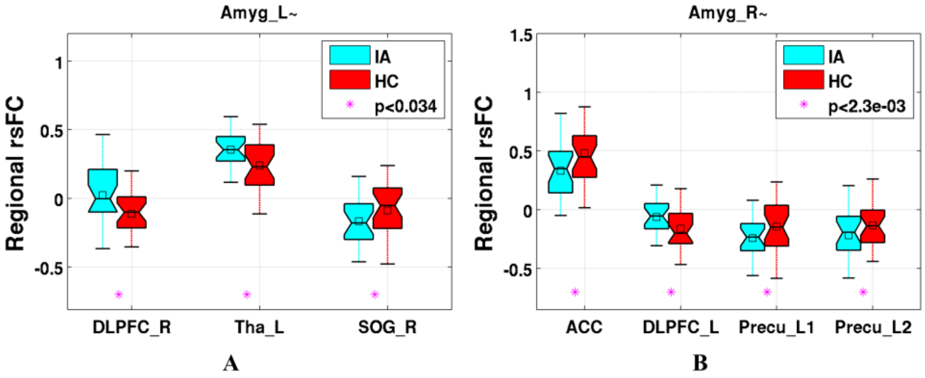

Supplementary Figure S2. Regional rsFC measures were calculated based on rsfMRI data pre-processed with the global signal regression, while the PFC regions were identified based on rsfMRI data without the global signal regression. For each box plot, the central mark is the median, the edges of the box are the 25th and 75th percentiles, and the whiskers extend from each edge of the box to the most extreme values within 1.5 times the interquartile range. Stars indicate that the difference was statistically significant ($p<0.05$). The abbreviations are given in Fig. 1 and Table 2.
